# Synthesis, Structure and Dye Adsorption Properties of Wine-Rack-Type Supramolecular Macrocycles Based on Polyoxovanadate

**DOI:** 10.3390/molecules30092075

**Published:** 2025-05-07

**Authors:** Nasen Bate, Baoshan Hou, Hongmei Gan

**Affiliations:** Xinjiang Key Laboratory of Clean Conversion and High Value Utilization of Biomass Resources, School of Chemistry and Chemical Engineering, Yili Normal University, Yining 835000, China; 04012@ylnu.edu.cn

**Keywords:** polyoxovanadate, self-assembly, supramolecular macrocycle, wine rack, dye adsorption

## Abstract

The precise construction and programmable assembly of structures with specific topologies remain persistent challenges in crystal engineering, primarily constrained by the limited availability of building blocks. Utilizing a synergistic approach that combines an in situ-formed concave polyoxovanadate (POV) cluster {VV_4_} with specifically designed 120° ditopic carboxylic acid bridging ligands, we successfully synthesized a series of wine-rack-type supramolecular macrocycles characterized by the general formula [(V_5_O_9_Cl)_4_(L)_8_]^8−^. The experimental results demonstrate that the introduction of sulfonic acid groups enables controlled structural extension into 1D chain and 2D layer architectures, manifesting the unique advantages of POV-based wine-rack units in constructing framework-based porous materials. This work significantly contributes to the structural diversity of wine-rack-type supramolecular architectures while simultaneously highlighting the great potential of polyoxometalate-driven supramolecular assemblies in materials science.

## 1. Introduction

The construction of supramolecular macrocyclic complexes represents a pivotal research area in supramolecular chemistry and coordination chemistry [1,2,3,4,5,6], attracting considerable attention in recent years due to these structures’ remarkable application potential across diverse fields such as catalysis, sensing, drug delivery, and materials science [7,8,9,10]. Among these, supramolecular macrocycles featuring “wine-rack” architectures have emerged as a prominent focus of investigation owing to their unique geometric configurations and distinctive mechanical properties. Current research efforts predominantly concentrate on elucidating the structural characteristics and functional behaviors of metal–organic frameworks and covalent organic frameworks incorporating wine-rack motifs [11,12]. However, the systematic design of supramolecular macrocycles with intrinsic wine-rack topologies remains underexplored.

Polyoxometalates (POMs) represent a prominent class of discrete anionic metal–oxo clusters, primarily constructed through corner-, edge-, or face-sharing configurations of early transition metal polyhedra (M = Mo, W, V, Nb, Ta, etc.) [13]. Among these, POV clusters stand out as exceptional building blocks for supramolecular architectures, owing to the pronounced polarization capability of V ions toward terminal oxygen atoms, which facilitates the formation of directional bowl-shaped subunits—a critical feature for assembling closed supramolecular architectures [14,15,16,17]. Pioneering studies by Michael’s group, Wang’s group, and others have systematically demonstrated that {V_4_} and {VV_4_} clusters are ideal for constructing capsule-like and octahedral structures [18,19,20], while {V_5_} and {MV_5_} (M = W, Mo, V, Nb) motifs exhibit high compatibility with icosahedral architectures [21,22]. Similarly, {V_6_} and {VV_6_} units are tailored for tetrahedral and cubic assemblies [23,24,25]. Notably, Navarro’s group and Hartl’s group have exemplified the versatility of {V_3_} clusters in constructing both tetrahedral and cubic superstructures [26,27], underscoring the structural adaptability of V-oxo clusters in supramolecular design.

In 2015, Michael’s group laid the foundation for wine-rack-type supramolecular macrocycles by constructing three pioneering structures based on {V_4_} clusters [28]. Herein, we demonstrate that wine-rack-type supramolecular macrocycles with the general formula [(V_5_O_9_Cl)_4_(L)_8_]^8−^, derived from {VV_4_} units, can be functionally controlled through the use of different vanadium sources and rigid angular ligands. A comparison of these two studies highlights the remarkable precision of atomic-level structural modulation achievable in supramolecular chemistry. By employing functionalized 120° dicarboxylic acid linkers for in situ assembly with {VV_4_} clusters, we successfully synthesized a series of wine-rack-type supramolecular macrocycles **(WR-VMOP-1–4)**. The sulfonic acid-functionalized one-dimensional (1D) and two-dimensional (2D) structures reveal the potential of POV-based wine-rack macrocycles as porous framework materials. POM crystalline materials can be used as solid adsorbents for adsorbing organic dyes [29,30,31]. Two main factors affect the adsorption process: one is the pore size that can accommodate the dye molecules, and the other is the role of ion exchange. Therefore, the dye adsorption properties for **WR-VMOP-3** were investigated.

## 2. Results and Discussion

### 2.1. Synthesis and Structure of Crystals

**Figure 1:**
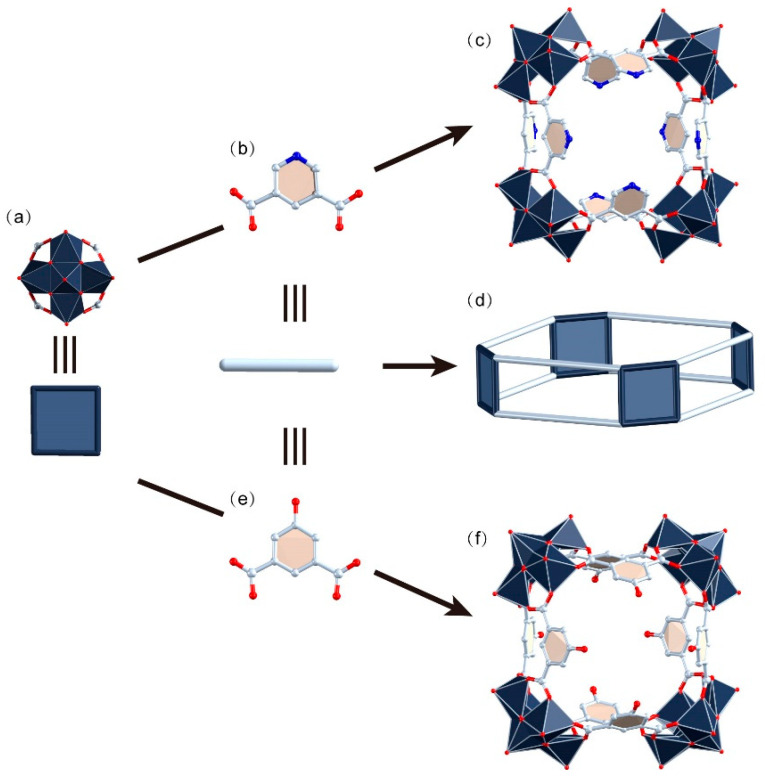
(**a**) Polyhedral representation of {VV_4_} units. (**b**) Ball-and-stick representation of 3,5-pyridinedicarboxylic acid. (**c**) Polyhedral representation of **WR-VMOP-1**. (**d**) Diagram of the wine-rack-type supramolecular macrocycles. (**e**) Ball-and-stick representation of 5-hydroxyisophthalic acid. (**f**) Polyhedral representation of **WR-VMOP-2**. Dark slate gray V, red O, pale blue C, blue N.

**WR-VMOP-1**, formulated as (NH_2_Me_2_)_12_(SO_4_)_4_[(V_5_O_9_Cl)_4_(1,3-bdc)_8_]·[solvents], was synthesized by reacting 3,5-pyridinedicarboxylic acid (Figure 1b) with VCl_4_ in a mixed solvent system of DMF, methanol, and acetonitrile at 130 °C for 2 days. Single-crystal X-ray diffraction (SCXRD) analysis reveals that **WR-VMOP-1** (Figure 1c) crystallizes in the monoclinic space group *C* 2/*c*. **WR-VMOP-1** comprises four pentanuclear [V_5_O_9_Cl(COO)_4_]^2−^ clusters (Figure 1a) interconnected by eight ligands, forming a wine-rack architecture (Figure 1d). The V^ᴠ^ centers are positioned above four basal V^ɪᴠ^ ions. The oxidation states of the vanadium cations are consistent with previous reports and further verified by bond valence sum calculations [32]. The apical V^ᴠ^ atom adopts a square-pyramidal geometry, coordinated by four *μ*_2_-O^2−^ anions and one terminal O^2−^ ligand. Each V^ɪᴠ^ center exhibits an octahedral coordination environment coordinated by two carboxylate groups, two *μ*_2_-O^2−^ ligands, one terminal O^2−^ ligand, and one *μ*_4_-Cl^−^ ligand. The V=O bond distances range from 1.573 to 1.604 Å, while V–O bond lengths vary between 1.876 and 2.027 Å (d_(V–Cl)_: 2.030–2.900 Å). The cavity of the structure features a Cl···Cl distance of 12.3 Å across the architecture (Figure S1). The dihedral angles between the phenyl rings of the ligands and the plane of the macrocycle range from 34° to 69°.

**WR-VMOP-2** was synthesized under conditions analogous to those for **WR-VMOP-1**, with the addition of VOSO_4_ and substitution of the 5-hydroxyisophthalic acid (Figure 1e) linker for 3,5-pyridinedicarboxylic acid. SCXRD analysis reveals that **WR-VMOP-2** (Figure 1f) crystallizes in the cubic space group *Pm-*3*m*. The architecture of **WR-VMOP-2** resembles that of **WR-VMOP-1**, except for a reduced window pore in **WR-VMOP-2** due to the steric influence of the hydroxyl group at the 5-position of the ligand. Notably, the crystal packing of **WR-VMOP-2** diverges significantly from that of **WR-VMOP-1**. In the c-axis direction of **WR-VMOP-1**, adjacent layers are separated by a wavy interlayer composed of sulfate anions and dimethylamine cations (Figure S2), resulting in non-overlapping molecular arrangements that preclude the formation of continuous channels. In contrast, for **WR-VMOP-2**, molecules along the c-axis are fully overlapped, creating unidirectional pore channels mirroring the intrinsic channel of individual macrocycles (Figure S3).

**Figure 2:**
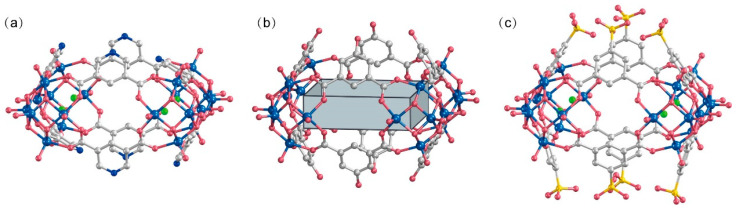
Molecule structure of (**a**) **WR-VMOP-1**, (**b**) **WR-VMOP-2**, (**c**) **WR-VMOP-3**. Royal blue V, Indian red O, grey C, blue N, bright green Cl.

**Figure 3:**
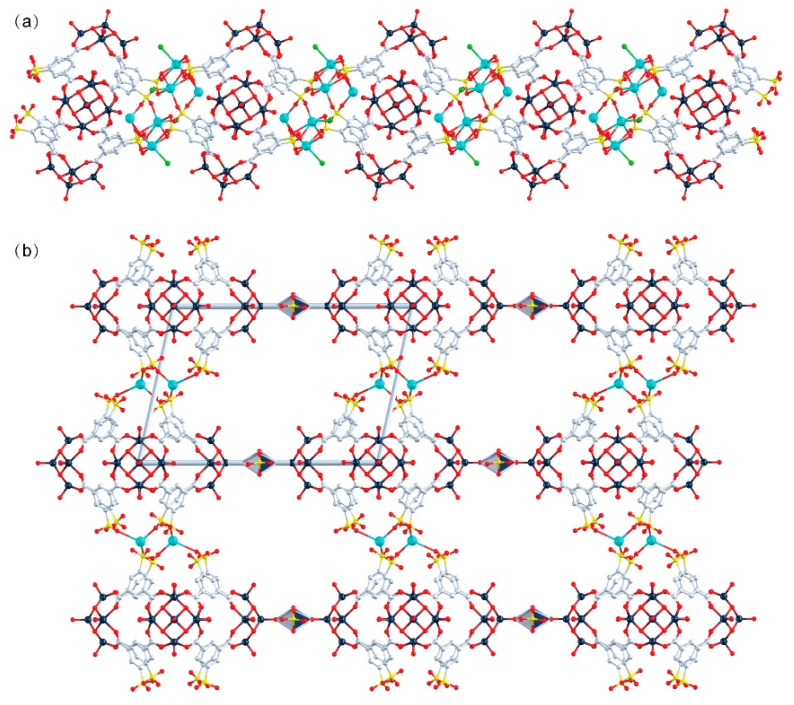
(**a**) One-dimensional structure of **WR-VMOP-3**, (**b**) Two-dimensional structure of **WR-VMOP-4**. Dark slate gray V, red O, pale blue C, yellow S, bright green Cl, turquoise Na.

The peripheral regions of the POVs clusters in **WR-VMOP-1** (Figure 2a) and **WR-VMOP-2** (Figure 2b) are enriched with oxygen atoms, rendering them highly suitable for coordinating with metal cations. This observation prompted us to explore the use of 5-sulfoisophthalic acid monosodium salt, a 120°-angled ligand bearing intrinsic sulfonate groups, to introduce additional coordination sites into the system. Employing the same synthetic protocol, we successfully obtained **WR-VMOP-3** (Figure 2c), a one-dimensional (1D) chain structure (Figure 3a) in which individual macrocycles retain the targeted wine-rack topology. Dihedral angles between the benzene rings and the macrocyclic plane (57–86°) confirm the structural flexibility of the macrocycle. Sodium ions coordinate with sulfonate groups to interconnect the macrocycles into 1D chains, while dimethylamine cations and sulfate anions balance the charge in the crystalline lattice. Crystallographic analysis reveals three distinct Na^+^ ions (Figure S4): one coordinates exclusively with three sulfonate groups within a single macrocycle, while the remaining two bridge adjacent macrocycles vertically to propagate the 1D chain. The Na–O bond distances range from 2.24 to 2.66 Å, and Na–Cl interactions are observed at approximately 2.67 Å. Substituting VCl_4_ with VOCl_3_ yielded **WR-VMOP-4**, a two-dimensional (2D) layered structure (Figure 3b), further demonstrating the structural extensibility of this wine-rack macrocycle series. In **WR-VMOP-4**, two Na^+^ ions link sulfate groups between vertically adjacent macrocycles to sustain the 1D chain, while vanadium ions bridge neighboring V_5_ clusters laterally to form a 2D network, creating parallelogram-shaped cavities bounded by four macrocycles. The bridging V center adopts a six-coordinate geometry (Figure S5) with V–O single bonds (1.95–2.15 Å), distinct from the V centers in the SBUs, which feature a V=O double bond. Comparative analysis highlights that **WR-VMOP-3** (1D) exhibits abundant Cl^−^ coordination sites, whereas **WR-VMOP-4** (2D) is enriched with sulfonate moieties. Both frameworks are classified as polyanionic architectures, underscoring their versatility in hosting diverse functional groups for targeted applications.

### 2.2. Dye Adsorption Study

**Figure 4:**
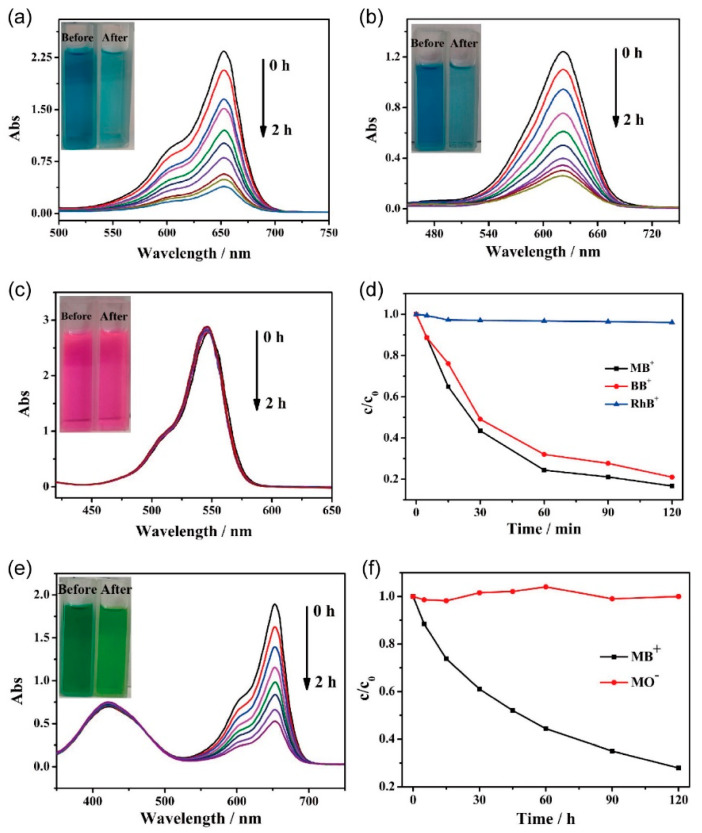
UV-vis adsorption spectra of methanol solution of (**a**) MB^+^, (**b**) BB^+^ and (**c**) RhB^+^ in **WR-VMOP-3**. (**d**) The change curve of the concentration of the dyes in the solution with time during the dyes adsorption process. (**e**) UV-vis adsorption spectra of the selective adsorption of **WR-VMOP-3** on mixed dye MB^+^/MO^−^. (**f**) The change curve of the concentration of dyes in the solution with time during the selective adsorption process.

Given the anionic nature of this series of wine-rack-type macrocycles, we selected **WR-VMOP-3** (Figures S6 and S7) to evaluate its dye adsorption properties. In the first phase, we compared the adsorption efficiency of **WR-VMOP-3** crystals toward dyes with similar molecular sizes but varying charges: methylene blue (MB^+^, cationic), methyl yellow (MY^0^, neutral), and methyl orange (MO^−^, anionic). Remarkably, the crystals exhibited strong adsorption affinity for the cationic dye MB^+^, achieving nearly complete adsorption within 2 h, as evidenced by the transition of the solution from intense blue to nearly colorless (Figure 4a). In contrast, negligible adsorption was observed for neutral MY^0^ and anionic MO^−^, with no significant changes in solution color (Figure S8), UV-vis absorption peak positions, or intensities after 2 h. These results demonstrate the inherent negative charge of **WR-VMOP-3**, which selectively adsorbs cationic dyes via electrostatic interactions.

In the subsequent phase, we investigated the size-dependent adsorption behavior of **WR-VMOP-3** toward cationic dyes by selecting three model dyes with identical single positive charge but varied molecular dimensions: rhodamine B (RhB^+^), brilliant blue (BB^+^), and methylene blue (MB^+^) (molecular size: RhB^+^ > BB^+^ > MB^+^). Remarkably, the crystals exhibited strong adsorption affinity for the smallest dye, MB^+^, achieving near-complete adsorption within 2 h, as evidenced by the transition of the solution from intense blue to nearly colorless (Figure 4a). For the medium-sized dye BB^+^, a moderate adsorption efficiency was observed, with a slight attenuation (Figure 4b) of the blue after 2 h. In contrast, negligible adsorption occurred for the largest dye, RhB^+^, as the solution retained its characteristic pink color (Figure 4c) throughout the experiment, with no discernible changes in UV-vis absorption spectra (Figure 4d). In general, as the size of the cationic dye is increased, the ion-exchange process becomes slower and slower. And steric constraints imposed by the macrocyclic framework also limit the size of the dyes that can be adsorbed. These results highlight the size-selective adsorption capability of **WR-VMOP-3**.

To further investigate the selective adsorption capability of **WR-VMOP-3** for mixed dyes with similar molecular dimensions, the crystals were immersed in a mixed solution containing MB^+^ and MO^−^. As shown in Figure 4e, the characteristic absorption peaks of MB^+^ exhibited rapid attenuation, while both the position and intensity of characteristic peak for MO^−^ remained essentially unchanged. This observation demonstrates that **WR-VMOP-3** can selectively capture the cationic dye MB^+^ from the mixed dyes system. Further verification was achieved by plotting the concentration ratio (C/C_0_) versus adsorption time (Figure 4f), where C_0_ represents the initial concentration. The relative concentration of MB^+^ showed a dramatic decline, whereas that of MO^−^ remained nearly constant at around 1 throughout the adsorption process. Minor fluctuations in MO^−^ concentration could be attributed to measurement errors during time-dependent sampling. These complementary analytical approaches collectively confirm the preferential adsorption behavior of **WR-VMOP-3** toward cationic dyes. After dye adsorption, **WR-VMOP-3** retained its crystalline integrity, as evidenced by the PXRD pattern (Figure S9).

## 3. Materials and Methods

All reagents were commercially sourced and utilized directly without additional purification. Note: VCl_4_ must be used in a fume hood.

### 3.1. Materials and Physical Techniques

All the reagents were obtained from commercial sources and used without further purification. Powder X-ray diffraction (PXRD) patterns were recorded ranging from 5 to 50° at room temperature on a Siemens D5005 diffractometer (Siemens AG, Munich, Germany) with Cu Kα (λ = 1.5418 Å). Thermogravimetric analysis (TGA) of the samples were performed using a Perkin–Elmer TG–7 analyzer (PerkinElmer Inc., Waltham, MA, USA) heated from room temperature to 800 °C under nitrogen at the heating rate of 10 °C·min^−1^. IR spectrum was performed in the range 4000–400 cm^−1^ using KBr pellets on an Alpha Centaurt FT-IR spectrophotometer (Bruker, Billerica, MA, USA).

### 3.2. X-Ray Crystallography

All data collections were performed on a Bruker D8–Venture diffractometer (Bruker) with a Turbo X-ray source (Cu K*α* radiation, *λ* = 1.5418 Å and Mo Kα radiation, λ = 0.71069 Å) adopting the direct drive rotating anode technique and a CMOS detector at 296 K. The data frames were collected using the program APEX 3 and processed using the program SAINT routine in APEX 3. The structures were solved by direct methods and refined by the full matrix least-squares on *F*^2^ using the SHELXL–2014 program. The diffused electron densities resulting from these residual solvent molecules were removed from the data set using the SQUEEZE routine of PLATON and refined further using the data generated. The restrained DFIX, SIMU, and ISOR instructions were used to make the structures more reasonable. We assigned a CCDC number of 2431104 for **WR-VMOP-1**, 2431105 for **WR-VMOP-2**, 2431106 for **WR-VMOP-3**, and 2431107 for **WR-VMOP-4**.

### 3.3. Dyes Adsorption Study

To evaluate the dye adsorption properties of **WR-VMOP-3**, five representative dyes were selected and tested in two phases. The dyes included methyl orange (MO^−^), which carries a single negative charge; methyl yellow (MY^0^), which is uncharged; and three dyes with a single positive charge but varying molecular sizes: rhodamine B (RhB^+^), basic blue 12 (BB^+^), and methylene blue (MB^+^). The molecular sizes of the cationic dyes follow the order RhB^+^ > BB^+^ > MB^+^, and the molecular structures of the dyes are illustrated in Figure S10. For the adsorption experiments, 20 mg of thoroughly washed and dried **WR-VMOP-3** was immersed in 5 mL of a dye/methanol solution with an initial concentration of 2.5 × 10^−5^ mol/L. To prevent any potential photochemical effects, the sample vials containing the mixtures were wrapped in aluminum foil and kept in the dark except during measurements. The changes in dye concentration were monitored by periodically measuring the absorbance of the dye solutions using a quartz cuvette with 3 cm length for ultraviolet–visible (UV-Vis) absorption spectroscopy. 

### 3.4. Synthesis of ***WR-VMOP-1***

3,5-Pyridinedicarboxylic acid (15 mg), DMF (2 mL), CH_3_OH (0.3 mL), CH_3_CN (0.2 mL), and VCl_4_ (4 drops) were sequentially added to a Parr Teflon-lined autoclave and kept at 130 °C for 48 h. After cooling down to room temperature, dark-green crystals were obtained and washed with CH_3_OH (yield: 30%, based on ligand). 

### 3.5. Synthesis of ***WR-VMOP-2***

5-Hydroxyisophthalic acid (15 mg), VOSO_4_ (20 mg), DMF (2 mL), CH_3_OH (0.3 mL), CH_3_CN (0.2 mL), and VCl_4_ (2 drops) were sequentially added to a Parr Teflon-lined autoclave and kept at 130 °C for 48 h. After cooling down to room temperature, dark-green crystals were obtained and washed with CH_3_OH (yield: 10%, based on ligand).

### 3.6. Synthesis of ***WR-VMOP-3***

5-Sulfoisophthalic acid monosodium salt (25 mg), VOSO_4_ (20 mg), DMF (2 mL), CH_3_OH (0.3 mL), CH_3_CN (0.2 mL), and VCl_4_ (2 drops) were sequentially added to a Parr Teflon-lined autoclave and kept at 130 °C for 48 h. After cooling down to room temperature, dark-green crystals were obtained and washed with CH_3_OH (yield: 45%, based on ligand).

### 3.7. Synthesis of ***WR-VMOP-4***

5-Sulfoisophthalic acid monosodium salt (20 mg), VOSO_4_ (20 mg), DMF (2 mL), CH_3_OH (0.3 mL), CH_3_CN (0.2 mL), and VOCl_3_ (2 drops) were sequentially added to a Parr Teflon-lined autoclave and kept at 130 °C for 48 h. After cooling down to room temperature, dark-green crystals were obtained and washed with CH_3_OH (yield: 5%, based on ligand).

## 4. Conclusions

In this study, we successfully constructed a series of wine-rack-type supramolecular macrocycles based on polyoxovanadates through the self-assembly of in situ-generated VV_4_ units and 120°-angled dicarboxylate ligands. Structural analyses reveal that the dihedral angles between the phenyl rings of the ligands and the macrocyclic plane vary from 34° to 86°, demonstrating significant conformational flexibility in these wine-rack-type macrocycles. The structural features of **WR-VMOP-4** particularly highlight the potential of extending the wine-rack motif into a framework structure. Furthermore, dye adsorption experiments were conducted to validate the anionic nature of this macrocycle series, providing experimental evidence for their potential applications in selective molecular recognition and separation processes.

## Data Availability

Data are contained within the article.

## Supplementary Materials

The following supporting information can be downloaded at: https://www.mdpi.com/article/10.3390/molecules30092075/s1, Table S1. The Crystallographic data for **WR-VMOP-1** and **WR-VMOP-2**. Table S2. The Crystallographic data for **WR-VMOP-3** and **WR-VMOP-4**. Figure S1. Molecule structure of **WR-VMOP-1**. Figure S2. Packing structure of **WR-VMOP-1**. Figure S3. Packing structure of **WR-VMOP-2**. Figure S4. Coordination mode of Na⁺ ions in **WR-VMOP-3**. Figure S5. Coordination mode of bridging V center in **WR-VMOP-4**. Figure S6. Packing structure of supramolecular macrocycles in **WR-VMOP-3**. Figure S7. Packing diagram of wine-rack macrocycles in **WR-VMOP-3**. Figure S8. UV-vis adsorption spectra of methanol solutions of (a) MY^0^ and (b) MO^−^ in **WR-VMOP-3**. Figure S9. PXRD pattern of **WR-VMOP-3** and **dye@WR-VMOP-3**. Figure S10. Structure formula of organic dyes. Figure S11. PXRD pattern of (a) **WR-VMOP-1,** (b) **WR-VMOP-3**, (c) **WR-VMOP-2**. Figure S12. IR curves of (a) **WR-VMOP-1,** (b) **WR-VMOP-3**, (c) **WR-VMOP-2**. Figure S13. TG curves of (a) **WR-VMOP-1,** (b) **WR-VMOP-3**, (c) **WR-VMOP-2**. Figure S14. IR (a) and TG (b) curves of **WR-VMOP-4**.
