# Supplementary material for: Synthesis, Structure and Dye Adsorption Properties of Wine-Rack-Type Supramolecular Macrocycles Based on Polyoxovanadate
Source: Molecules. 2025 May 7;30(9):2075. doi: 10.3390/molecules30092075 (PMC12073448; doi:10.3390/molecules30092075)
Supplement: Supplementary file 1 [file molecules-30-02075-s001.zip › molecules-3558362-supplementary.pdf]

Supporting Information

Synthesis, Structure and Dye Adsorption Properties of Wine-rack-type Supramolecular Macrocycles based on Polyoxovanadate

Nasen Bate, Baoshan Hou* and Hongmei Gan *

Xinjiang Key Laboratory of Clean Conversion and High Value Utilization of Biomass Resources, School of Chemistry and Chemical Engineering, Yili Normal University, Yining, Xinjiang, 835000, China.

*Corresponding author

E-mail: ganhm@jlu.edu.cn (H.-M. Gan)

E-mail: houbaoshan@westlake.edu.cn (B. S. Hou)

Table S1. The Crystallographic data for **WR-VMOP-1** and **WR-VMOP-2**

Compound	**WR-VMOP-1**	**WR-VMOP-2**
Empirical formula	$C_{60}H_{40}Cl_4N_{10}O_{84}S_4V_{20}$	$C_{64}H_{32}Cl_4O_{76}V_{20}$
Formula weight	3533.86	3177.61
Crystal system	Monoclinic	Cubic
Space group	*C2/c*	*Pm*-3*m*
Temperature (K)	296.15	296.15
Wavelength (Å)	0.71073	0.71073
a (Å)	39.122(7)	29.473(4)
b (Å)	17.472(3)	29.473(4)
c (Å)	25.168(4)	29.473(4)
α (°)	90	90
β (°)	109.233(3)	90
γ (°)	90	90
Volume ($Å^3$)	16243(5)	25602(10)
Z	4	4
Dcalc. /mg·m-3	1.445	0.824
μ/mm^{-1}	1.292	0.780
F(000)	6936.0	6200.0
Limiting indices	-46<=h<=37, -20<=k<=20, -28<=l<=29	-32<=h<=32, -32<=k<=32, -23<=l<=32
Theta range for data collection (°)	2.888 to 50.138	1.382 to 46.938
Reflections collected	44936	126487
Independent reflections	14295 [R(int) = 0.0692]	3681 [R(int) = 0.4524]
Refinement method	Full-matrix least-squares on F^2	Full-matrix least-squares on F^2
Data/restraints/parameters	14295/1704/822	3681/0/164
Goodness-of-fit on F^2	1.032	1.014
Final R indices [I> 2sigma(I)]	R_1 = 0.0841, wR_2 = 0.2322	R_1 = 0.0795, wR_2 = 0.2336
R indices (all data)	R_1 = 0.1383, wR_2 = 0.2842	R_1 = 0.1512, wR_2 = 0.2998

Table S2. The Crystallographic data for **WR-VMOP-3** and **WR-VMOP-4**

Compound	**WR-VMOP-3**	**WR-VMOP-4**
Empirical formula	$C_{75}H_{65}Cl_8N_5Na_6O_{100}S_{10}V_{20}$	$C_{68}H_{40}Cl_4N_2NaO_{100}S_{100}V_{21}$
Formula weight	4397.26	4040.15
Crystal system	Monoclinic	Monoclinic
Space group	*C2/c*	*C2/c*
Temperature (K)	292.5	296.05
Wavelength (Å)	0.71073	1.54178
a (Å)	23.597(4)	31.012(5)

b (Å)	35.831(6)	26.101(4)
c (Å)	31.262(4)	29.759(5)
α (°)	90	90
β (°)	102.160(5)	109.985(9)
γ (°)	90	90
Volume ($Å^3$)	25839(7)	22638(6)
Z	4	4
Dcalc. /mg·m-3	1.130	1.185
μ/mm^{-1}	0.924	8.856
F(000)	8688.0	7936.0
Limiting indices	-27<=h<=25, -42<=k<=42, -36<=l<=36	-35<=h<=35, -30<=k<=30, -35<=l<=35
Theta range for data collection (°)	4.6 to 49.51	4.544 to 130.162
Reflections collected	151693	70463
Independent reflections	22053 [R(int) = 0.0682]	18641[R(int)= 0.1078]
Refinement method	Full-matrix least-squares on F^2	Full-matrix least-squares on F^2
Data/restraints/parameters	22053/2095/1062	18641/1788/938
Goodness-of-fit on F^2	1.024	1.057
Final R indices [I > 2sigma(I)]	R_1 = 0.0638, wR_2 = 0.1871	R_1 = 0.1277, wR_2 = 0.2511
R indices (all data)	R_1 = 0.0945, wR_2 = 0.2168	R_1 = 0.1814, wR_2 = 0.3904

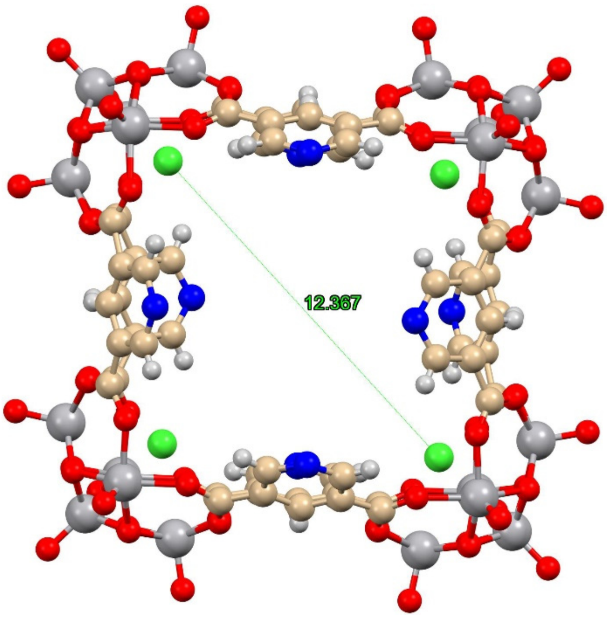

Figure S1 Molecule structure of **WR-VMOP-1**.

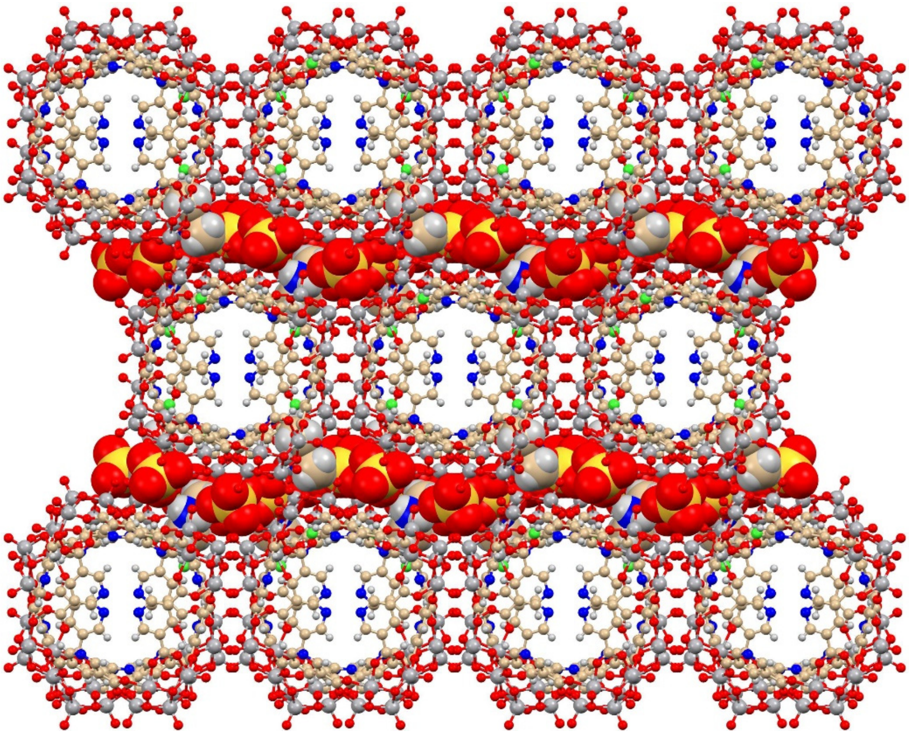

Figure S2 Packing structure of **WR-VMOP-1**.

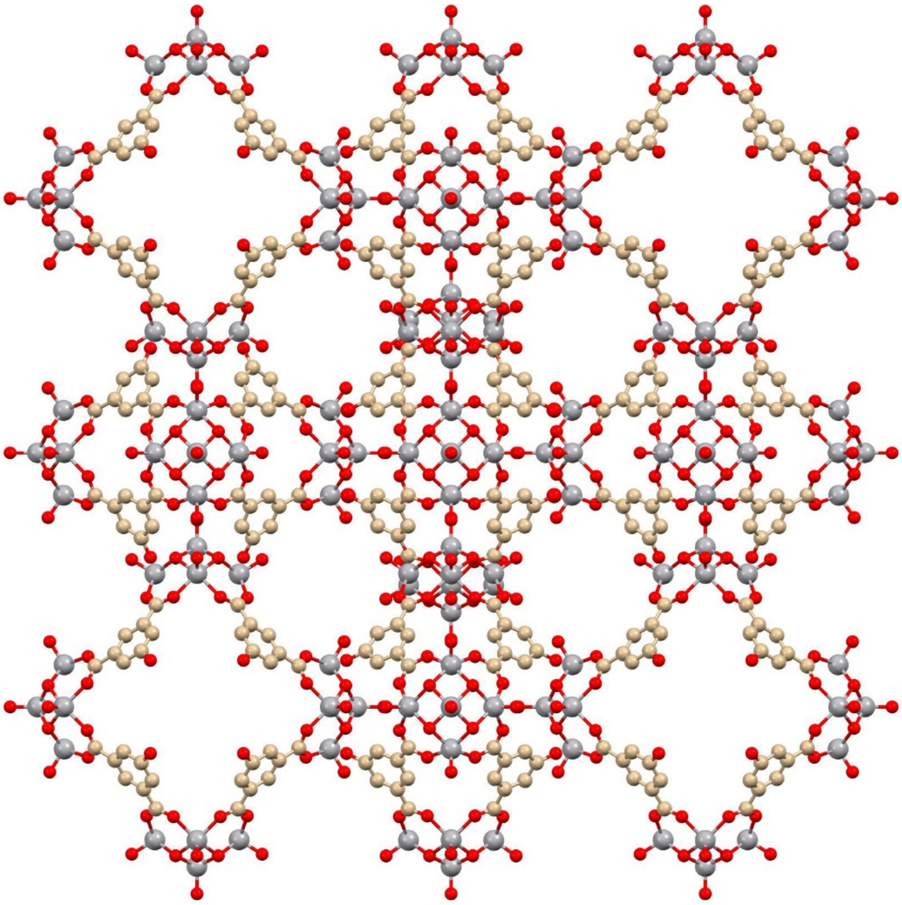

Figure S3 Packing structure of **WR-VMOP-2**.

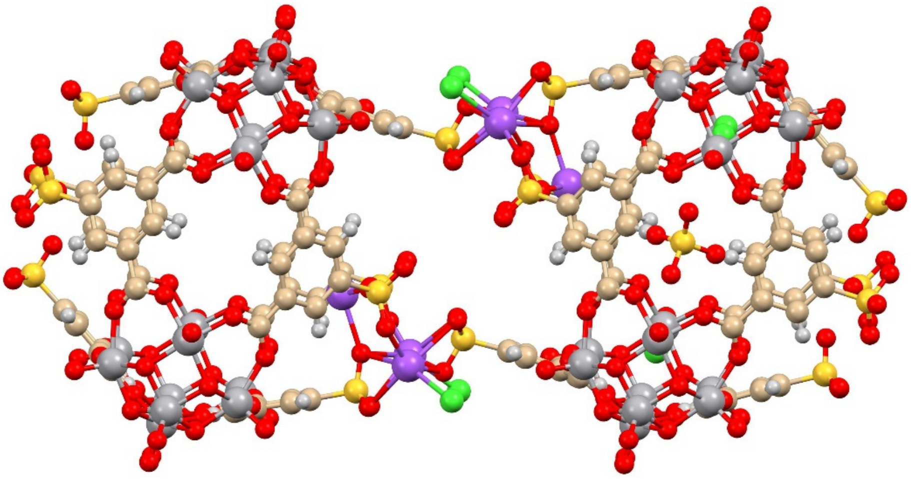

Figure S4 Coordination mode of Na^+ ions in **WR-VMOP-3**.

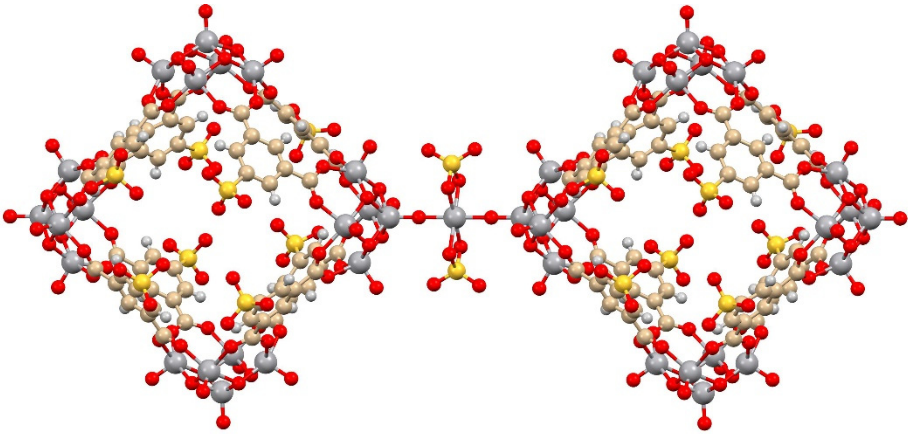

Figure S5 Coordination mode of bridging V center in **WR-VMOP-4**.

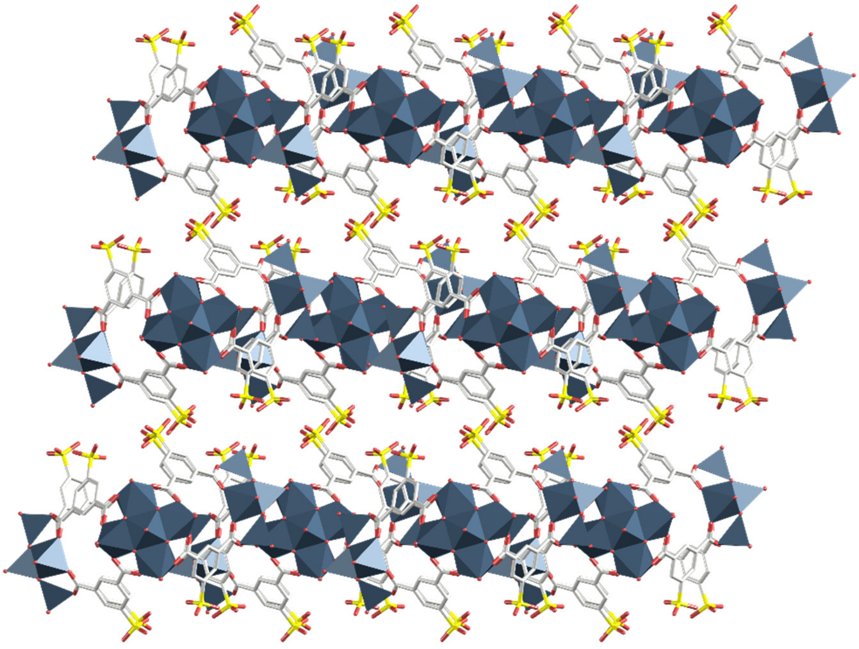

Figure S6 Packing structure of supramolecular macrocycles in **WR-VMOP-3**.

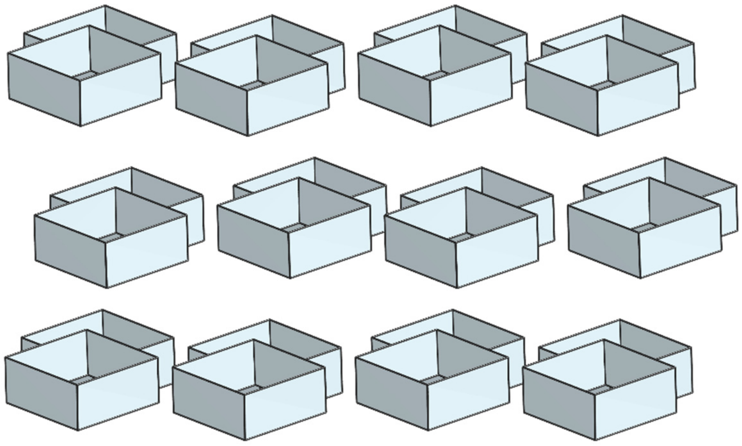

Figure S7 Packing diagram of wine-rack macrocycles in **WR-VMOP-3**.

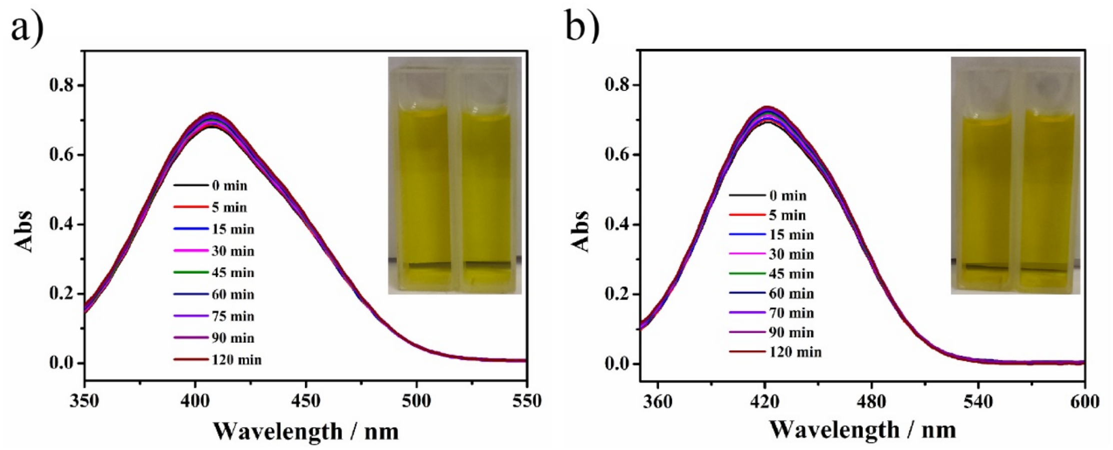

Figure S8 UV-vis adsorption spectra of methanol solutions of (a)MY^0 and (b)MO^- in **WR-VMOP-3.**

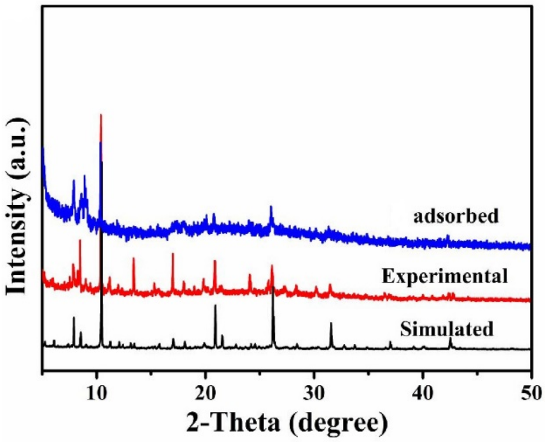

Figure S9 PXRD pattern of **WR-VMOP-3** and **dye@WR-VMOP-3**.

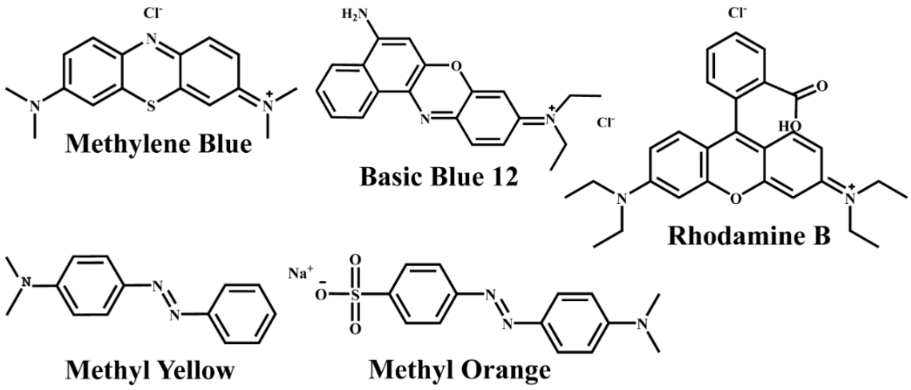

Figure S10 Structure formula of organic dyes.

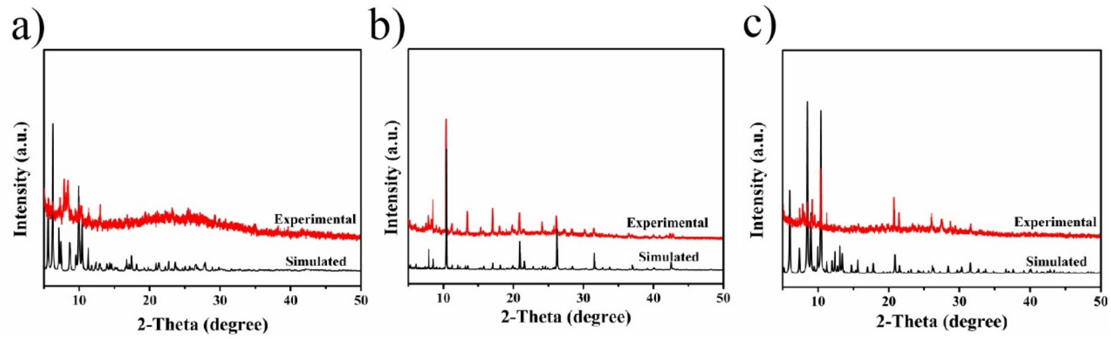

Figure S11 PXRD pattern of (a) **WR-VMOP-1,** (b) **WR-VMOP-3**, (c) **WR-VMOP-2**.

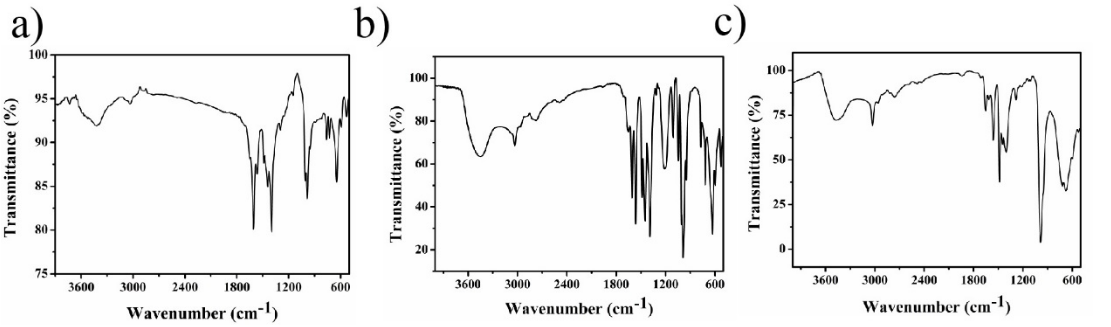

Figure S12 IR curves of (a) **WR-VMOP-1,** (b) **WR-VMOP-3**, (c) **WR-VMOP-2**.

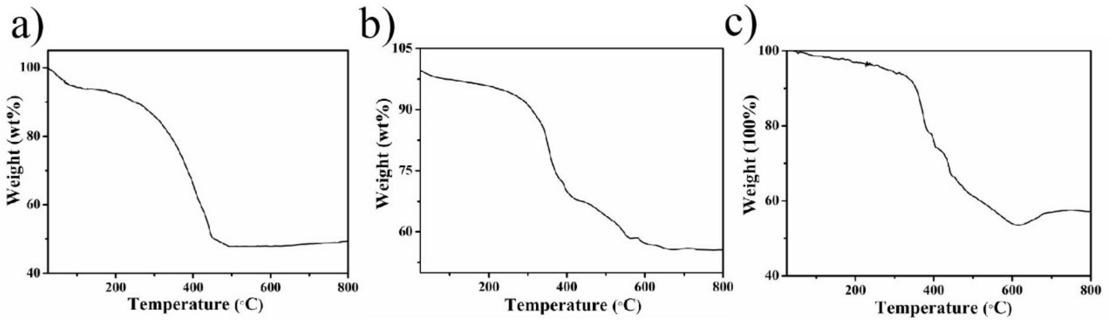

Figure S13 TG curves of (a) **WR-VMOP-1,** (b) **WR-VMOP-3**, (c) **WR-VMOP-2**.

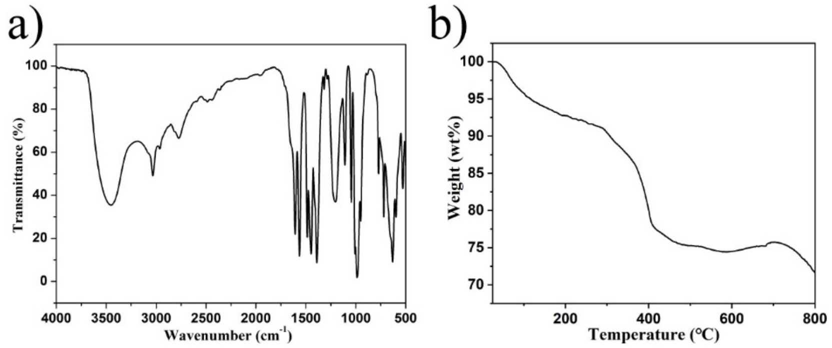

Figure S14 IR (a) and TG (b) curves of **WR-VMOP-4**.

As for the FT-IR spectrum of **WR-VMOP-1-4**, 500-1200cm^{-1} region contains the absorption band of metal-oxygen tensile vibration, which usually corresponds to the characteristic region of polyoxometalates. The two the characteristic peaks at about 715-763cm^{-1} are attributed to the V-O-V bridging segments. The peaks around 970-1138cm^{-1} are associated with SO_4^{2-} in the structures. The characteristic peaks at 1560-1600cm^{-1} are due to the carboxylic acid in ligands, while the peaks near about 800cm^{-1} and 2900cm^{-1} are related to the C-H bond of the benzene ring. Finally, the peaks band at 3000-3500 cm^{-1} indicates the presence of $[(CH_3)_2NH_2]^+$ cations in these structures.

TG analysis in N_2 of **WR-VMOP-1-4** exhibit similar continuous weight loss process. The first weight loss range from 25 °C to 260-310 °C attribute to the lost of solvent molecules (MeOH + CH_3CN + DMF). WR-VMOPs can maintain good thermal stability at least until 250 °C, while the skeleton will collapse as the temperature continues to rise.
